# LPAR6 Inhibits the Progression of Hepatocellular Carcinoma (HCC) by Suppressing the Nuclear Translocation of YAP/TAZ

**DOI:** 10.3390/ijms26094205

**Published:** 2025-04-29

**Authors:** Gegentuya Bao, Manjue Zhai, Yali Yan, Yuewu Wang, Alatangaole Damirin

**Affiliations:** School of Life Sciences, Inner Mongolia University, Hohhot 010110, China; 15754830927@163.com (G.B.);

**Keywords:** lysophosphatidic acid receptor, HCC, cell proliferation, cell migration, signaling pathway

## Abstract

Lysophosphatidic acid (LPA), a key bioactive lipid, modulates cellular functions through interactions with LPA receptors (LPAR1-6) of the G protein-coupled receptor (GPCR) family, participating in both physiological and pathological processes. While LPA/LPAR signaling typically promotes cancer progression by regulating angiogenesis and cancer cell metastasis, our study unexpectedly reveals that LPA exhibits an inhibitory effect on cellular activity in hepatocellular carcinoma (HCC). We further investigate the specific receptor subtypes mediating these effects and elucidate the underlying mechanisms at the cellular, tissue, and organismal levels. Pharmacological studies demonstrated that LPA predominantly inhibits HCC progression through activation of LPAR6. Mechanistically, LPA/LPAR6 activation suppresses HCC proliferation, migration, and epithelial–mesenchymal transition (EMT). In vivo, LPAR6 overexpression in a nude mouse xenograft model significantly reduced tumor growth rate and volume, accompanied by decreased Ki-67 expression in tumor tissues, as shown by immunohistochemical analysis. Transcriptomic analysis combined with Western blot experiments demonstrated that LPA/LPAR6 inhibits YAP/TAZ nuclear translocation, thereby suppressing HCC cell proliferation and migration. In conclusion, these findings suggest that enhancing LPAR6 expression or developing LPAR6 agonists may offer a promising therapeutic strategy for adjuvant cancer treatment.

## 1. Introduction

According to the latest global cancer statistics, hepatocellular carcinoma (HCC) remains one of the most prevalent cancers worldwide [1]. Over the past decade, both the incidence and mortality rates of HCC have steadily increased, drawing widespread attention from health sectors around the world [2,3]. The main causes include exposure to aflatoxins, non-alcoholic fatty liver disease (NAFLD), hepatitis B and C infections, and excessive alcohol consumption [4,5]. Currently, treatment options for HCC, including surgery, interventional therapies, and chemotherapy, have shown limited effectiveness, with a persistently low five-year survival rate [6]. In recent years, PD-1/PD-L1 inhibitors (such as pembrolizumab and nivolumab) have demonstrated positive effects in treating HCC, particularly in advanced-stage patients [7,8]. However, in subsequent clinical trials, monotherapy with various antibodies failed to meet the primary survival endpoints [9]. As a result, it is crucial to discover novel molecular targets and develop combination immunotherapy strategies to enhance the effectiveness of treatments for patients with hepatocellular carcinoma (HCC). Identifying these new therapeutic targets, along with synergistic treatment approaches, could significantly improve patient outcomes by addressing the complexity and resistance mechanisms inherent in HCC.

Lysophosphatidic acid (LPA) is a bioactive lipid-signaling molecule primarily produced during platelet activation, and it is also secreted by fibroblasts and tumor tissues [10]. Currently, six LPA receptors (LPAR1-6, with non-human receptors denoted as Lpar1-6) have been identified, each coupled to different G proteins and responsible for a range of biological functions [11]. These functions mainly involve regulating cell proliferation, differentiation, motility, secretion, and microenvironment formation, while also participating in various physiological and pathological processes [12] such as disease progression and drug resistance [13]. All LPA receptors are part of the G protein-coupled receptor (GPCR) family, making them key targets in GPCR structural biology and small molecule drug research [14,15]. It is well-known that GPCRs form the largest superfamily of membrane proteins and are crucial targets in drug development [16]. As part of the GPCR family, LPA receptors have become a major focus of drug research due to their pivotal roles in various physiological and pathological processes [11]. Studying the structure, function, and interactions of LPA receptors is of great academic and practical importance for developing targeted small-molecule drugs and improving disease treatment.

In recent years, our research has conducted an in-depth investigation into the expression patterns of lysophosphatidic acid receptors (LPARs) across different tissues and cell types, with a particular focus on tumor tissues. Our findings indicate that the expression profiles of these receptors vary significantly between different tissue types and cell subpopulations. This tissue- and cell-specific expression suggests that LPARs may play distinct roles in various physiological and pathological processes, particularly in the context of cancer, where their differential expression could influence tumor progression and response to treatment. Additionally, different LPARs are coupled with distinct types of G proteins in various tissues and cells, activating specific signaling pathways to perform diverse regulatory functions. For example, LPAR1 shows high expression in lung cancer tissues, making it a potential target for novel lung cancer treatment strategies [17]. LPAR2 facilitates renal cancer progression mainly by activating the MAPK and NF-κB pathways [18]. Existing studies have demonstrated that LPA levels are significantly elevated in the ascites of ovarian cancer patients [19]. Our further studies indicated that the LPA-induced oncogenic effects in ovarian cancer cells involve the LPAR3/Gi/MAPKs/NF-κB signaling pathway [20]. Notably, our recent findings reveal that LPA can suppress the proliferation and migration of HCC. We are especially interested in identifying the receptor responsible for mediating this inhibitory effect of LPA.

Therefore, this study aims to identify the specific LPA receptor subtype involved in inhibiting HCC proliferation using pharmacological and molecular biology methods. Stable cell lines with subtype-specific receptor expression will be established to investigate the receptor’s role, signaling mechanisms, and their implications in tumor-bearing animal models and transcriptomic analyses. Additionally, the potential of this receptor subtype as a molecular target for HCC therapy will be evaluated. In conclusion, our research not only deepens the understanding of how bioactive lipids regulate cellular functions and their underlying mechanisms from a fundamental theoretical perspective but also provides new insights into developing small-molecule drugs in the field of medical applications. This work could offer a novel molecular target for HCC treatment.

## 2. Results

### 2.1. Expression Pattern and Immune Correlation of LPAR6 Based on Multi-Database Analysis

**Figure 1:**
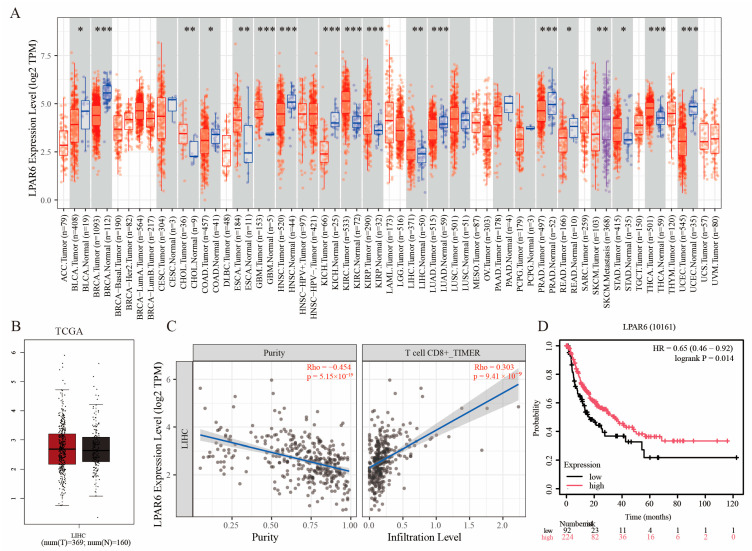
Analysis of the Expression Pattern, Immune Relevance, and Prognostic Significance of LPAR6 in LIHC. (**A**) Differential expression of LPAR6 across various cancer types based on analysis from the TIMER2.0 database. (**B**) Comparison of LPAR6 mRNA expression levels between LIHC tissues and normal liver tissues using integrated TCGA and GTEx datasets. (**C**) Correlation between LPAR6 expression and immune cell infiltration in LIHC. (**D**) Kaplan–Meier survival analysis of overall survival in LIHC patients based on LPAR6 expression levels. * p < 0.05; ** p < 0.01; *** p < 0.001.

Genes that are abnormally expressed in tumor tissues are often closely associated with tumor initiation and progression. Through analysis using the TIMER2.0 database, we found that, compared to corresponding normal tissues, LPAR6 expression was downregulated in 10 different cancer types and significantly upregulated in 8 other cancers (Figure 1A). Further comparison based on the TCGA database combined with GTEx normal tissue data revealed no significant difference in LPAR6 mRNA expression levels between liver hepatocellular carcinoma (LIHC) tissues and normal tissues (Figure 1B).

Interestingly, immune infiltration analysis via the TIMER platform showed a significant negative correlation between LPAR6 expression and tumor purity, suggesting that high LPAR6 expression may be closely associated with infiltration of non-tumor components such as immune cells. Further correlation analysis indicated that LPAR6 expression was positively associated with infiltration levels of various immune cells, particularly CD8^+^ T cells, implying that LPAR6 may be involved in regulating antitumor immune responses and shaping the tumor immune microenvironment. In addition, Kaplan–Meier survival analysis demonstrated that patients with high LPAR6 expression had significantly better overall survival compared to those with low expression.

### 2.2. LPA Inhibits the Activity of Hepatocellular Carcinoma (HCC) Cells Through the LPAR6 Receptor

**Figure 2:**
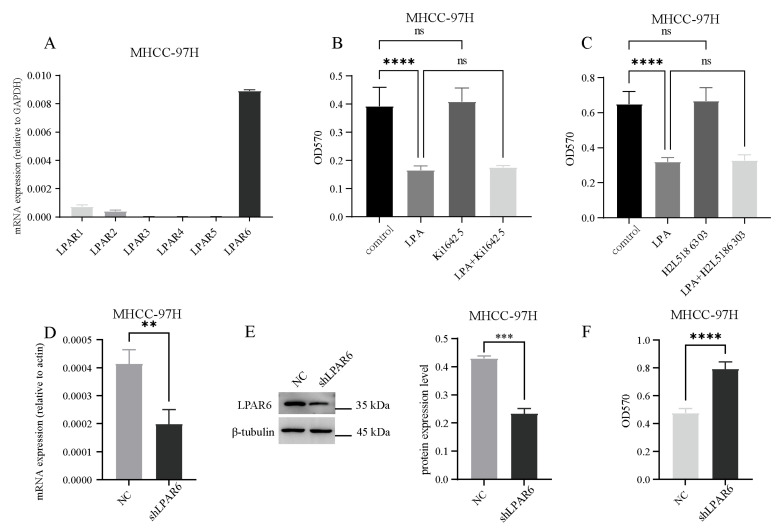
LPAR6 inhibits HCC cell proliferation. (**A**) The expression levels of six LPARs were measured using real-time PCR. (**B**,**C**) Cells were pretreated with 1 µM Ki16425 or 1 µM H2L5186303, followed by stimulation with or without 1 µM LPA. Cell proliferation activity was assessed by the MTT assay. (**D**) The knockdown efficiency of LPAR6 was evaluated using real-time PCR. (**E**) Western blotting was used to assess the knockdown efficiency of LPAR6. (**F**) The cell viability was assessed by the MTT assay after LPA stimulation. ns, not significant; ** p < 0.01; *** p < 0.001; **** p < 0.0001.

LPA regulates different signaling pathways through the activation of its six receptors (LPAR1-6), thereby inducing various cellular responses and functions. Therefore, we examined the expression profile of LPA receptors in HCC cells. As shown in Figure 2A, LPAR1, LPAR2, LPAR3, and LPAR6 were expressed to varying degrees. To determine the primary LPA receptor involved in the inhibition of HCC cell viability, we treated HCC cells with pharmacological agents: Ki16425 (a selective antagonist of LPAR1 and LPAR3) and H2L5186303 (a selective inhibitor of LPAR2) and assessed their cell viability. Compared to the control group (Figure 2B,C), LPA significantly inhibited HCC cell viability, while the addition of Ki16425 and H2L5186303 had no effect on cell viability. These results suggest that LPAR1-3 may not be key receptors in LPA-induced inhibition of HCC cell viability. Next, we constructed transgenic cells with LPAR6 interference (Figure 2D,E). As shown in Figure 2F, cell viability was significantly increased after shLPAR6 infection. This result is consistent with our hypothesis and previous findings that LPAR6 plays an inhibitory role in HCC cell viability. Silencing LPAR6 with shRNA relieves this inhibitory effect, thereby promoting cell viability. This finding highlights the functional role of LPAR6 in regulating HCC cell viability.

### 2.3. LPA Inhibits HCC Cell Proliferation, Migration, and EMT via LPAR6

**Figure 3:**
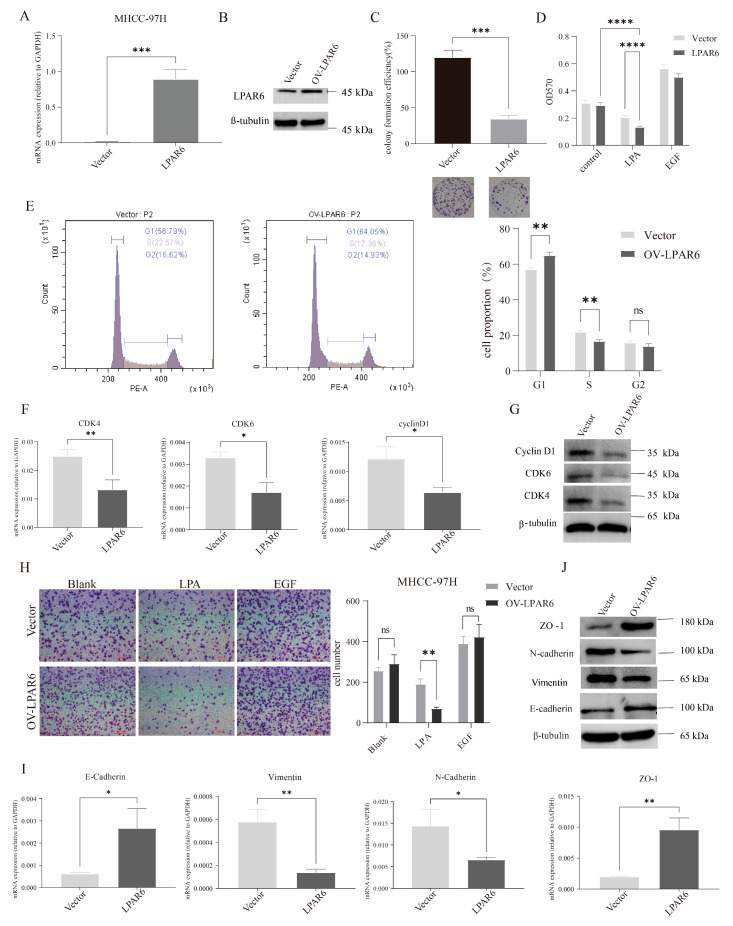
The impact of the LPA/LPAR6 axis on HCC cell functions. (**A**) LPAR6 mRNA expression in MHCC-97H-Vector and MHCC-97H-LPAR6 cells was measured using real-time PCR. (**B**) LPAR6 protein expression in both cell lines was assessed by Western blotting. (**C**) Overexpression of LPAR6 inhibited colony formation in MHCC-97H cells. Magnification: ×100. (**D**) Cell proliferation was evaluated using the MTT assay. Cells were treated with 1 µM LPA, with 25 ng/mL EGF as a positive control. (**E**) Cell cycle analysis was performed to determine the percentage of cells in different phases. Cells were stained with propidium iodide and analyzed by flow cytometry. (**F**) Real-time PCR was used to measure the expression of cell cycle-related genes. (**G**) Western blot was used to detect the expression of cell cycle-related proteins. (**H**) The ability of cells to migrate was assessed using Transwell chambers, with cells cultured under 1 µM LPA stimulation for 4 h. The scale bar represents 200 μm. (**I**) Real-time PCR was used to detect the mRNA expression of EMT-related genes in MHCC-97H-Vector and MHCC-97H-LPAR6 cell. (**J**) Western blotting was performed to evaluate the protein expression of EMT-related markers in both cell lines. ns, not significant; * p < 0.05; ** p < 0.01; *** p < 0.001; **** p < 0.0001.

To explore the role and mechanism of LPAR6 in HCC more deeply, we successfully overexpressed LPAR6 in HCC cells by lentiviral transfection (Figure 3A,B). Overexpression of LPAR6 inhibited clone formation in MHCC-97H cells (Figure 3C). Cell proliferation assay results showed (Figure 3D) that LPA stimulation significantly inhibited the proliferation of cells overexpressing LPAR6. These results are consistent with our previous knockdown experiments, indicating that LPAR6 plays a key role in the inhibition of HCC cell proliferation. Abnormal cell cycle regulation is closely related to tumorigenesis and progression. To investigate the role of LPAR6 in the HCC cell cycle, we used flow cytometry to analyze the cell cycle distribution in the vector and LPAR6 overexpression groups. As shown in Figure 3E, overexpression of LPAR6 increased the proportion of cells in the G0/G1 phase, while the number of cells in the S phase was significantly reduced. This suggests that LPAR6 slowed down the transition of MHCC-97H cells from G1 to S phase and ultimately inhibited cell proliferation. The cell cycle is regulated by a variety of cell cycle proteins, and aberrant expression or loss of these proteins disrupts normal cycle progression [21]. As shown in Figure 3F,G, overexpression of LPAR6 significantly inhibited the expression of cell cycle-related proteins. This suggests that LPAR6 inhibits cell proliferation by regulating the expression of cell cycle-associated proteins such as CDK4, CDK6, and Cyclin D1.

Enhanced cell migration is a marker of tumor malignancy [22], and the results of our cell migration assay similarly showed that LPAR6 overexpression significantly reduced cell migration (Figure 3H). Epithelial–mesenchymal transition (EMT) is closely related to tumor cell migration, invasion, metastasis, and drug resistance [23]. We found that overexpression of LPAR6 significantly inhibited the expression of the mesenchymal markers’ vimentin and N-cadherin, while promoting the expression of the epithelial markers’ tight junction protein ZO-1 and E-cadherin (Figure 3I,J).

### 2.4. The LPA/LPAR6 Axis Can Suppress the Occurrence of Tumors In Vivo

**Figure 4:**
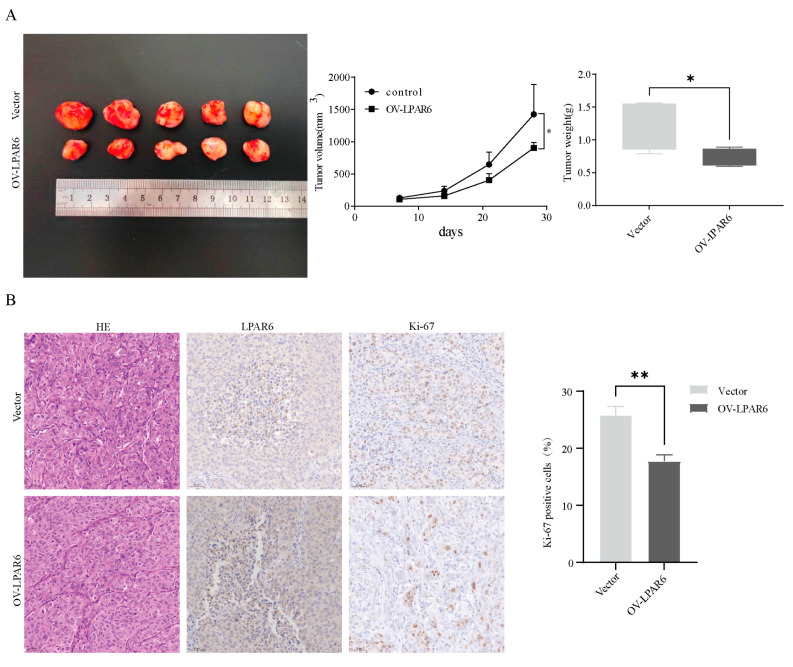
LPAR6 inhibits tumor growth. (**A**) Tumor images in the transgenic model: Vector and LPAR6 overexpression cells were subcutaneously injected into the left and right flanks of mice (2 × 10^7^ cells per injection). Tumor volume and weight were measured. (**B**) Immunohistochemical detection of LPAR6 and Ki-67 expression in MHCC-97H xenografts (20×). * p < 0.05; ** p < 0.01.

To confirm the consistency between the in vitro results and the in vivo role of LPAR6, we established a human liver cancer cell xenograft model in nude mice, extending the study to an animal experiment level. The results showed that the average tumor volume in the LPAR6 overexpression group was significantly smaller in nude mice, and weekly tumor volume measurements indicated that LPAR6 overexpression significantly slowed tumor growth. From the third week after cell inoculation, differences in tumor growth rate and volume became evident (Figure 4A). At the tissue level, immunohistochemical staining was used to assess the expression of Ki-67 and LPAR6. Ki-67 is a marker of proliferating cells, with higher positive rates indicating faster tumor growth. Compared to the control group, tumor samples from the LPAR6 overexpression group showed reduced Ki-67 expression and increased LPAR6 expression (Figure 4B). Through in vivo experiments, we observed that LPAR6 plays a positive role in inhibiting tumor initiation and progression, further supporting its potential as a therapeutic target.

### 2.5. The Hippo Signaling Pathway Is Involved in LPAR6-Mediated Inhibition of HCC Cell Proliferation and Migration

**Figure 5:**
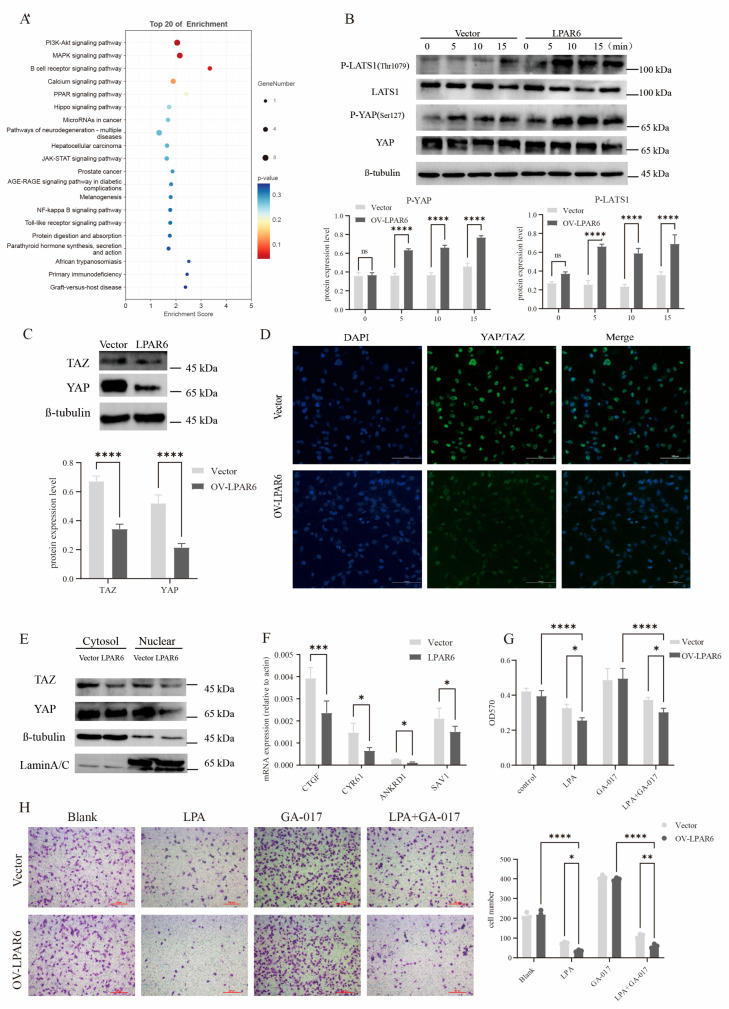
The Hippo signaling pathway plays a role in LPA/LPAR6-mediated inhibition of cell proliferation and migration. (**A**) KEGG enrichment analysis was performed to assess pathway involvement. (**B**) MHCC-97H cells were transfected with either an empty vector or LPAR6 overexpression construct, followed by short-term stimulation with 1 µM LPA for 0, 5, 10, and 15 min. Western blot analysis was then performed to assess the phosphorylation levels of LATS1 and YAP. (**C**) Western blot was performed to detect the protein expression levels of YAP and TAZ in total cell lysates after stimulation with 1 µM LPA for 6 h. (**D**) Immunofluorescence staining was performed to visualize YAP/TAZ localization. Scale bar = 100 μm. (**E**) Protein levels of YAP and TAZ were assessed in the nuclear and cytoplasmic fractions with Western blot analysis. (**F**) Real-time PCR was used to measure the mRNA expression levels of downstream target genes in the Hippo signaling pathway. (**G**,**H**) Cell proliferation and migration activity were measured after pretreatment with GA-017. The scale bar represents 200 μm. ns, not significant; * p < 0.05; ** p < 0.01; *** p < 0.001; **** p < 0.0001.

To investigate the impact of LPAR6 on HCC, we performed transcriptomic analysis on control and LPAR6 overexpressing cells after LPA stimulation. KEGG pathway enrichment analysis was used to identify the pathways enriched with differentially expressed genes. The results showed (Figure 5A) that the differentially expressed genes were predominantly enriched in the PI3K-AKT, MAPK, and Hippo signaling pathways. The Hippo signaling pathway plays a crucial role in regulating cell proliferation, tissue development, and apoptosis. To further explore whether the Hippo pathway is involved in LPAR6-mediated cell function, we stimulated vector and LPAR6 overexpressing cells with LPA for various durations and assessed the phosphorylation levels of the key kinase LATS1 and its main effector YAP. The results showed (Figure 5B) that, in the LPAR6 overexpression group, the phosphorylation levels of LATS1 and YAP were significantly increased. In addition, LPA stimulation for 6 h led to a significant upregulation of LPAR6, which in turn inhibited the expression of YAP and TAZ (Figure 5C). YAP/TAZ (Yes-associated protein/Transcriptional co-activator with PDZ-binding motif) are central effectors of the Hippo signaling pathway, involved in signal transduction and gene transcription regulation, ultimately influencing cell proliferation, growth, and fate [24]. Western blot and immunofluorescence confocal analysis revealed that LPAR6 overexpression reduced the expression of YAP and TAZ in both the cytoplasm and nucleus (Figure 5D,E), consequently inhibiting the transcription of downstream Hippo pathway target genes such as CYR61, CTGF, ANKRD1, and SAV1 (Figure 5F). These results suggest that LPAR6 activates the Hippo signaling pathway, thereby inhibiting the nuclear translocation of YAP/TAZ. GA-017 is a potent selective inhibitor of LATS1 and LATS2 (large tumor suppressor kinases 1/2), which promotes the nuclear translocation of YAP/TAZ and acts as a cell proliferation activator [25]. In vitro experiments showed that, compared to the control group, LPAR6 significantly inhibited HCC cell proliferation and migration, while GA-017 alone promoted these processes. However, the combination of LPA and GA-017 stimulation counteracted the cell proliferation and migration induced by GA-017, with the LPAR6 overexpression group showing a more pronounced effect (Figure 5G,H). These results indicate that the Hippo signaling pathway is involved in LPA/LPAR6-mediated inhibition of HCC cell proliferation and migration.

## 3. Discussion

The discovery of lysophosphatidic acid (LPA) represents a significant milestone in the field of lipid biology. Initially, it was identified through studies investigating the effects of cytokines or growth factors on cells. Researchers first noted the presence of a substance in some extracellular fluids and biological materials that could activate cellular responses, which was later identified as LPA. Subsequent studies gradually elucidated LPA’s physiological roles in cell proliferation, migration, and morphological changes [26]. As research progressed, the identification of LPA receptors (LPAR1-6) further highlighted the importance of LPA signaling pathways in cellular biology and pathology [27]. These receptors are coupled with various G proteins, activating multiple downstream signaling pathways and mediating diverse cellular responses. These findings provided a crucial foundation for exploring LPA’s roles in diseases, particularly cancer. It is noteworthy that each LPA receptor exhibits distinct functions in the motility, invasion, and proliferation of cancer cells, further highlighting the complexity of LPA in tumor biology.

LPAR1-3 belong to the EDG subfamily of the GPCR family and are widely expressed in cells, making them the most extensively studied receptors to date [28]. In contrast, LPAR4-6 receptors, which are non-EDG members of the GPCR family, are less broadly expressed, and their functional roles remain poorly understood in many aspects [29]. However, in recent years, as research on LPAR1-3 has deepened, it has been recognized that the regulatory functions induced by LPA result from the combined effects of all LPA receptors expressed in cells. Consequently, the contributions and influences of each receptor type cannot be ignored, leading to a growing interest in studying LPAR4-6 (non-EDG family receptors). The focus of this study, LPAR6, is a non-EDG subfamily member of the GPCR family [30]. In 2009, Yanagida et al. [31] identified the orphan receptor P2Y5 as the sixth receptor for lysophosphatidic acid and elucidated the existence of the LPA-LPAR6-Rho signaling pathway. Prior to this, Pasternack et al. [32] published a paper in Nature Genetics reporting that the P2Y5 receptor, specifically expressed in hair follicle cells, plays a role in hair development and that P2Y5 polymorphisms are associated with hair thinning and alopecia in humans. Shortly thereafter, Lee et al. [33] reported that the LPAR6/P2Y5 receptor expressed in the intestinal mucosa is coupled with Gi or G12/13 proteins, reducing cell–cell adhesion in the mucosal epithelium. Mazzocca et al. [34] reported in Cancer Research that the LPAR6 receptor exhibits tumor-promoting properties, arguing that LPAR6 facilitates tumor mass formation, which is partially attributed to enhanced adhesion between tumor cells. This finding contradicts previous reports suggesting that LPAR6 reduces intercellular adhesion. In contrast, in breast cancer, LPAR6 acts as a tumor suppressor [35,36]. We observed that LPAR6 and other LPA receptors exhibit strong tissue- and cell-specificity. Furthermore, these receptors can couple with different G proteins, leading to distinct cellular functions. Therefore, research in this field urgently needs to clarify the “double-edged” commonality and specificity of LPA receptors to provide more precise conclusions, laying a theoretical foundation for applied studies.

In our study, we observed that LPA broadly inhibits the proliferation and migration of HCC. Transcriptomic analysis revealed 905 differentially expressed genes (DEGs) in the LPAR6 high-expression group compared to the vector group, with 367 genes upregulated and 538 genes downregulated (Figure S1). KEGG pathway analysis of these DEGs revealed significant enrichment in signaling pathways such as PI3K-AKT, MAPK, and Hippo. These pathways are closely associated with key biological processes such as cell proliferation, differentiation, and apoptosis, and may play an important role in the onset and progression of the disease. Although this study reveals that LPA/LPAR6 inhibits HCC cell proliferation and migration through the Hippo signaling pathway, it does not explore the interaction between LPA/LPAR6 and other cell signaling pathways (such as PI3K-AKT, MAPK, etc.), which could provide insight into how different signaling pathways collaborate to regulate tumor progression. Currently, research on the role of LPAR6 in HCC and related cancers is still limited, and further in-depth studies are needed to explore the potential and mechanisms of this receptor. In summary, by combining transcriptomics, we explored the function and mechanisms of LPAR6 at the cell, tissue, and animal levels, and found that LPAR6 acts as a tumor-suppressive receptor in hepatocellular carcinoma. Therefore, increasing the expression of LPAR6 receptors and developing LPAR6 agonists will undoubtedly provide a new approach for adjunctive cancer therapy.

However, the current study is based on a single HCC cell line, and using a single cell line may not fully reflect the cellular behaviors and responses in different types of liver cancer. Therefore, the findings of this study may have certain limitations. To further validate the function of LPAR6 and expand the scope of the research, future studies should consider using multiple HCC cell lines and further evaluate its role in in vivo models. Additionally, the heterogeneity of cell lines and the impact of the tumor microenvironment may also play a significant role in the research results. Therefore, increasing the diversity of cell lines and considering the factors of the tumor microenvironment will contribute to a more comprehensive understanding of the role of LPAR6 in liver cancer.

## 4. Materials and Methods

### 4.1. Cell Culture

The human hepatocellular carcinoma cell line MHCC-97H was purchased from Procell Life Science & Technology Co., Ltd. (Wuhan, Hubei, China). All cell lines were cultured in Dulbecco’s Modified Eagle’s Medium (DMEM) (BI) containing 10% fetal bovine serum (BI), with incubation conditions of 37 °C, 20% O_2_, and 5% CO_2_. The RNA interference sequences are listed in Table S1.

### 4.2. Cell Proliferation and Colony Formation

Cell proliferation ability was assessed using the Solarbio MTT Assay Kit (Cell Proliferation Detection Kit; Solarbio, Beijing, China). Cells were seeded into a culture plate and cultured overnight in serum-free medium after attachment. The medium was then replaced with the stimulating solution for 24 h, followed by the addition of an appropriate amount of MTT solution. The cells were incubated for 2–4 h, and the absorbance was recorded using a microplate reader (Bio-Tek, Winooski, VT, USA). Cells were seeded in 6-well plates at a low density and cultured for approximately 10–14 days under normal conditions, with medium changes every 2–3 days. When visible colonies formed, the cells were fixed with 4% paraformaldehyde for 15 min and stained with 0.1% crystal violet for 30 min. The number of colonies (≥50 cells) was counted manually under a light microscope (Nikon Eclipse Ti, Nikon Corporation, Tokyo, Japan).

### 4.3. Cell Cycle Analysis and Cell Migration

Cells were harvested and fixed in 70% ethanol at 4 °C overnight. Following fixation, the cells were washed twice with PBS and incubated with RNase A (100 µg/mL) and propidium iodide (PI, 50 µg/mL) at 37 °C for 30 min in the dark. DNA content was analyzed by flow cytometry using a BD FACSCanto II flow cytometer (BD Biosciences, San Jose, CA, USA). The distribution of cells in G0/G1, S, and G2/M phases was determined using FlowJo software (version 10.8.1, FlowJo LLC, Ashland, OR, USA). Cell migration was also assessed using Transwell chambers (8-µm pore size; Tree Star Inc., Ashland, OR, USA). Cells were suspended in serum-free medium and seeded into the upper chamber, while the lower chamber was filled with medium containing 10% FBS as a chemoattractant. After incubation for 24 h at 37 °C, non-migrated cells on the upper surface of the membrane were removed with a cotton swab. The migrated cells on the lower side were fixed with 4% paraformaldehyde, stained with 0.1% crystal violet, and counted under a microscope in five randomly selected fields.

### 4.4. Cell Transfection

Stable overexpression cell lines were constructed using lentiviral vectors. To produce the virus, pHBLV-CMV-MCS-EF1-Luc-T2A-Puro and pHBLV-CMV-MCS-EF1-Luc-T2A-Puro-LPAR6 were co-transfected with the packaging plasmid psPAX2 and the envelope plasmid pMD2.G into HEK293 cells using Lipofectamine 2000 transfection reagent (Thermo Fisher Scientific, Waltham, MA, USA), according to the manufacturer’s instructions. After 24 h, the medium was replaced, and the supernatant was collected 48 h later. MHCC-97H cells were infected with lentivirus-vector (MHCC-97H-vector) or lentivirus-LPAR6 (MHCC-97H-LPAR6). After puromycin (Sangon Biotech, Shanghai, China) selection, the expression at both RNA and protein levels was validated.

### 4.5. RNA Extraction and RT-qPCR

The cell status was observed under a microscope. After confirming appropriate cell density and morphology, the culture medium was carefully aspirated and discarded. An appropriate amount of RNAiso Plus (Takara, Shiga, Japan) was added to the cells to lyse them. The dish was gently shaken to ensure even distribution of the reagent and thorough lysis of the cells. The cell lysate was then transferred into a centrifuge tube and pipetted up and down several times to homogenize the solution until no visible precipitate remained. An appropriate volume of chloroform was added to the lysate. The tube was tightly closed and shaken vigorously for a few seconds until the mixture turned milky white, indicating complete emulsification. The tube was then centrifuged at 12,000× *g* for 10 min at 4 °C. After centrifugation, the solution separated into three distinct phases: a colorless upper aqueous phase (containing RNA), a white interphase, and a pink lower organic phase. The upper aqueous phase was carefully transferred to a new centrifuge tube, and an equal volume of isopropanol was added. The tube was inverted several times to mix thoroughly, then centrifuged at 12,000× *g* for 10 minutes at 4 °C. A white RNA pellet was typically visible at the bottom of the tube. The supernatant was gently removed without disturbing the pellet. To wash the RNA, 75% ethanol (pre-cooled) was added to the pellet, followed by gentle inversion to mix. The sample was centrifuged again at 7500× *g* for 5 min at 4 °C. The supernatant was discarded, and the pellet was air-dried at room temperature. Finally, the RNA pellet was dissolved in an appropriate amount of RNase-free water. RNA concentration and purity were assessed using a spectrophotometer. Using RNA as a template, first-strand cDNA was synthesized in the same reaction tube with the TransScript^®^ RT/RI Enzyme Mix (TransGen Biotech, Beijing, China), while gDNA Remover was added to remove any residual genomic DNA from the RNA template. The corresponding reagents were added on ice and incubated for 15 min. After the reaction, the mixture was heated at 85 °C for 5 s to simultaneously inactivate both TransScript^®^ RT and gDNA Remover. The remaining steps are as follows [37]. The primer sequences are provided in Supplementary Table S2.

### 4.6. Western Blot Analysis

Total protein was extracted from cells using RIPA lysis buffer containing protease and phosphatase inhibitors. Protein concentrations were quantified using a BCA Protein Assay Kit (Solarbio, Beijing, China). Equal amounts of protein samples (20–40 µg) were separated by SDS-PAGE and transferred onto PVDF membranes. After blocking with 5% non-fat milk at room temperature for 1 h, the membranes were incubated overnight at 4 °C with the following primary antibodies: LPAR6 (Cell Signaling Technology, Danvers, MA, USA), CDK4 (Bioss, Beijing, China), CDK6 (Bioss, Beijing, China), Cyclin D1 (Bioss, Beijing, China), vimentin (Bioss, Beijing, China), ZO-1 (Bioss, Beijing, China), E-cadherin (Bioss, Beijing, China), N-cadherin (Bioss, Beijing, China), β-tubulin (TransGen Biotech, Beijing, China), YAP (Cell Signaling Technology, Danvers, MA, USA), phosphorylated YAP (p-YAP, Cell Signaling Technology, Danvers, MA, USA), TAZ (Cell Signaling Technology, Danvers, MA, USA), LATS1 (Cell Signaling Technology, Danvers, MA, USA), phosphorylated LATS1 (p-LATS1, Cell Signaling Technology, Danvers, MA, USA), and LaminA/C (TransGen Biotech, Beijing, China). After washing, the membranes were incubated with HRP-conjugated secondary antibodies for 1 h at room temperature. Protein bands were visualized using enhanced chemiluminescence (ECL) reagents and imaged using a chemiluminescence imaging system (ChemiScope series, Qinxiang Scientific Instruments Co., Ltd., Shanghai, China).

### 4.7. Xenograft Models

Six-week-old male BALB/c nude mice were purchased from SPF (Beijing, China) Biotechnology Co., Ltd. MHCC-97H-vector or MHCC-97H-LPAR6 cells were subcutaneously injected into the right and left flank regions of each mouse. Tumor volumes were measured every ten days. The mice were euthanized on days 30 to 60 after cell injection, and tumor weight and volume were measured.

### 4.8. RNA Sequencing

RNA was extracted from the control group and LPAR6-overexpressing cells after 6 h of LPA stimulation as described above. Sequencing was performed on an Illumina platform by OE Biotech Co., Ltd. (Shanghai, China).

### 4.9. H&E Stain, Immunohistochemistry (IHC), and Cell Immunofluorescence

Tissue samples were fixed in 4% paraformaldehyde, embedded in paraffin, and cut into 4 μm thick sections. After deparaffinization and rehydration, the sections were stained with hematoxylin for 5 min, rinsed, and counterstained with eosin for 2 min. The stained sections were dehydrated, cleared, mounted, and observed under a light microscope.

Paraffin-embedded tissue sections were deparaffinized, rehydrated, and subjected to antigen retrieval by heating in citrate buffer (pH 6.0). Endogenous peroxidase activity was blocked with 3% hydrogen peroxide. The sections were then incubated with primary antibodies at 4 °C overnight, followed by HRP-conjugated secondary antibodies for 1 h at room temperature. Visualization was performed using DAB substrate, and nuclei were counterstained with hematoxylin.

Cells were seeded onto glass coverslips and fixed with 4% paraformaldehyde for 15 min. After permeabilization with 0.1% Triton X-100, cells were blocked with 5% BSA for 1 h and incubated with primary antibodies at 4 °C overnight. After washing, cells were incubated with fluorophore-conjugated secondary antibodies for 1 h at room temperature in the dark. Nuclei were counterstained with DAPI. Fluorescent images were captured using a fluorescence microscope (model Eclipse Ni-E, Nikon Corporation, Tokyo, Japan).

### 4.10. Statistical Analysis

Statistical and graphical data analyses were performed using GraphPad Prism 7 software (version 7.0, GraphPad Software Inc., San Diego, CA, USA). The data are presented as mean ± S.E.M. from at least three independent experiments. The analysis was performed using one-way and two-way ANOVA, as well as the *t*-test. A value of *p* < 0.05 was considered statistically significant.

## Data Availability

The data presented in this study are available on request from the corresponding author.

## Supplementary Materials

The following supporting information can be downloaded at: https://www.mdpi.com/article/10.3390/ijms26094205/s1.
