# Supplementary material for: LPAR6 Inhibits the Progression of Hepatocellular Carcinoma (HCC) by Suppressing the Nuclear Translocation of YAP/TAZ
Source: Int J Mol Sci. 2025 Apr 29;26(9):4205. doi: 10.3390/ijms26094205 (PMC12071778; doi:10.3390/ijms26094205)
Supplement: Supplementary file 1 [file ijms-26-04205-s001.zip › ijms-3539728-supplementary.pdf]

Supplementary Materials

Figure S1 : Differentially expressed genes

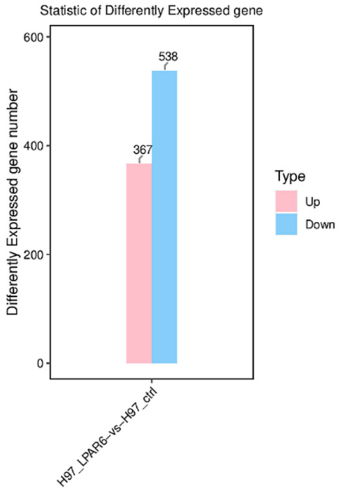

Bar chart of differentially expressed genes

Table S1: RNA interference sequence

Negative Control:5'-ATCGACTAGCCACTTAGAC-3'

shLPAR6:5'-GGGAAGGAAAGGAAAGAAA-3'

Table S2: Sequences of primers used for real-time PCR.

Name	Forward primer sequence 5'- 3'	Reverse primer sequence5'- 3'
LPAR1	TCCTTCGTCAGGGCCTCATT	ACCGTAATGTGCCTCTCGATT
LPAR2	GCTTCCACCAGCCCATCTAC	TGAGGAAGAGGTAGGCCACG
LPAR3	GCAACCTGACCAAAAAGAGGG	GCAATTCCAGCCCAGTGTG
LPAR4	TCCTTACCAACATCTATGGGAGC	ACGTTTGGAGAAGCCTTCAAAG
LPAR5	ACTCGGTGGTGAGCGTGTA	GTGCAGTGCGTAGTAGGAGA
LPAR6	CACCCGCCGTTTTTGTT CAG	ATGTTTTCCATGTGGCTTCTGG
CDK4	GATGGAAGTGCCTCCGGAT	GATCCAGTGAGGCTTCCTGA
CDK6	CTGACCAGCAGTACGAAGCTG	TGACGAAGTGGGTCTTGACG
CYCLIND1	CTGACTGCAAACTGCGAGGAG	GGGTGGGTTGGAAATGAACTTGA
CTGF	CCAATGACAACGCCTCCTG	TGGTGCAGCCAGAAAGCTC
CYR61	AGCCTCGCATCCTATACAACC	TTCTTTCACAAGGCGGCACTC
ANKRD1	CACTTCTAGCCCACCCTGTGA	CCACAGGTTCCGTAATGATTT
SAV1	ATCAACCACCTCCTGTCA	GCAACTTTAGCATTCCCT
ACTIN	GCACTCTTCCAGCCTTCCTT	AATGCCAGGGTACATGGTGG
GAPDH	GAA GGT GAA GGT CGG AGT C	GGA GAT GGT GAT GGG ATT TC
